# A High-Performance Supercapacitor Based on Hierarchical Template-Free Ni/SnO_2_ Nanostructures via Hydrothermal Method

**DOI:** 10.3390/ma17081894

**Published:** 2024-04-19

**Authors:** Abdul Samad Shameem, Anbazhagan Murugan, Vadivel Siva, Govindasamy Palanisamy, Ikhyun Kim, Jintae Lee, Sivaprakash Paramasivam

**Affiliations:** 1 Department of Science and Humanities, Karpagam Academy of Higher Education, Coimbatore 641 021, India; 2 Centre for Energy and Environment, Karpagam Academy of Higher Education, Coimbatore 641 021, India; 3 Department of Science and Humanities, Karpagam College of Engineering, Coimbatore 641 032, India; 4 Department of Physics, Karpagam Academy of Higher Education, Coimbatore 641 021, India; 5 School of Chemical Engineering, Yeungnam University, 280 Daehak-Ro, Gyeongsan 38541, Republic of Korea; 6 Department of Mechanical Engineering, Keimyung University, Daegu 42601, Republic of Korea

**Keywords:** Ni/SnO_2_, hydrothermal method, specific capacitance, supercapacitor, cycling stability

## Abstract

Novel flake-like Ni_1−x_Sn_x_O_2_ particles were successfully prepared by template-free hydrothermal synthesis. The prepared samples were investigated for their properties by different characterization techniques. Scanning micrographs showed that the obtained particles consisted of nanoflakes. The X-ray diffraction results of the Ni_1−x_Sn_x_O_2_ revealed the formation of mixed-phase Ni/SnO_2_ having the typical tetragonal structure of SnO_2,_ and the cubic structure of Ni in a nanocrystalline nature. The doping with Ni had a certain influence on the host’s lattice structure of SnO_2_ at different doping concentrations. Confirmation of the functional groups and the elements in the nanomaterials was accomplished using FTIR and EDS analyses. The electrochemical performance analysis of the prepared nanomaterials were carried out with the help of the CV, GCD, and EIS techniques. The specific capacitance of the synthesized nanomaterials with different concentrations of Ni dopant in SnO_2_ was analyzed at different scanning rates. Interestingly, a 5% Ni-doped SnO_2_ nanocomposite exhibited a maximum specific capacitance of 841.85 F g^−1^ at 5 mV s^−1^ in a 6 M KOH electrolyte. Further, to boost the electrochemical performance, a redox additive electrolyte was utilized, which exhibited a maximum specific capacitance of 2130.33 at 5 mV s^−1^ and an excellent capacitance retention of 93.22% after 10,000 GCD cycles. These excellent electrochemical characteristics suggest that the Ni/SnO_2_ nanocomposite could be utilized as an electrode material for high-performance supercapacitors.

## 1. Introduction

Affordable, eco-friendly, and renewable energy resources and their consequent applications have prompted their strenuous pursuit due to the depletion of fossil energy sources and the effects of climatic change [1,2,3,4,5]. Subsequently, there has arisen an increased desire for energy resources that are sustainable and renewable. Nowadays, significant research is aimed at maintaining significant energy sustainability in this field. As part of the massive effort to accomplish this goal, supercapacitors, also known as ultracapacitors or electrochemical supercapacitors, have drawn a lot of curiosity owing to their valuable features, such as their protracted cyclic stability [6,7], superior power and energy densities [8], and their performance being considerably higher than that of other energy storage devices like faradic batteries, electrostatic capacitors, electrolytic capacitors, ceramic capacitors, etc. [9,10]. Therefore, dynamic research aimed at developing an efficient substitute material to achieve this goal is always predominant. So far, research has primarily focused on three classes of materials, namely, carbon, conducting polymers, and metal-based materials [11]. Carbonaceous materials are electric double-layer capacitors with significant surface area and porosity, which are highly conductive materials [12,13]. However, owing to their double-layer capacitance, carbon-based materials possess limited capacity, making it difficult to produce adequate energy to fulfill the rising demands of contemporary electronic devices [14]. Conductive polymers and metal-based materials are pseudocapacitors. These materials rely on fast faradaic reactions, which display higher capacitance and energy density than electric double-layer capacitors [13,15]. MXene–conducting polymers have garnered a lot of scientific interest in the field of energy storage. The electrochemical behavior of an MXene–PANI electrode revealed a specific capacitance of 430 F g^−1^, which is greater than both MXene–PPy (305 F g^−1^) and pristine MXene (105 F g^−1^), respectively [14]. Recently, Fan et al., established an electrode material for a supercapacitor utilizing the weak-base-assisted hydrolysis of Zn-MOF-74 accompanied by 850 ◦C carbonization to attain bundle-like carbon superstructures (SBC-850) as working electrodes with large surface areas and porosities, which achieved the highest specific capacitance of 270 F g^−1^ at 1 A g^−1^ in 1 M H_2_SO_4_ electrolyte in a three-electrode configuration [16]. The electrochemical performance was limited for conducting polymers maybe because of the characteristic volumetric shrinkage that occurs during the discharge of counterions [17].

The electrode material has a major impact on the performance of pseudocapacitors. To boost their energy/power ratio, supercapacitors need to be upgraded to satisfy the collective demands placed on them, such as through the use of novel, cost-effective, and ecologically sustainable electrode materials that must be designed and developed for practical use [7,13]. Transition metal chalcogenides, such as oxides, sulfides, selenides, and tellurides, have recently become the subject of intensive research on their potential for supercapacitor applications [18,19,20]. Researchers have considered g-CN as a suitable candidate owing to its higher capacitance and environmental friendliness. Hussain et al. synthesized a NiS@g-CN hybrid electrode via a hydrothermal technique and the experimental results revealed a remarkably high C_sp_ of 933.78 F g^−1^ at 1 A g^−1^, which was significantly higher than both individual pristine NiS and pristine g-CN electrodes. However, inferior electrical conductivity, insufficient ion diffusion, restricted capacity for high charge/discharge rates, electrode material disintegration, and deterioration of electrical connections within the active material are major problems commonly reported in regard to the weakened cyclic performance of nickel sulfide [21]. Metal oxides have emerged as a suitable material for supercapacitor electrodes as they exhibit a pseudocapacitive effect.

Among the transition metal oxides, RuO_2_ exhibits a high pseudocapacitance [22]. Yet, the replacement of RuO_2_ electrodes is required owing to its cost factor and environmental harmfulness. Recently, SnO_2_-based nanomaterials have garnered considerable interest as appealing electrode materials for supercapacitors and lithium-ion batteries owing to their lower cost and high power density [23]. Yet, their practical application remains limited by the poor transfer of electrolyte ions and poor electrical conductivity. In recent times, to further boost the electrochemical performance of metal oxides, various techniques such as the doping/incorporation of nitrogen, metals, metal oxides, sulfides, carbon-based materials, polymers, etc. are employed [4,9,11,23]. Among a variety of divalent dopants such as Cu^2+^, Co^2+^, Ni^2+^, and so on, Ni^2+^ has the same radius as tetravalent Sn^2+^. Nickel ions added into the structure of tin dioxide are expected to have an impact on the density of states of SnO_2_ while also causing minimal defect formation during the synthesis process, as compared to other divalent 3d dopants [24,25]. Ni has distinguished itself from other elements due to its excellent and promising properties, which include low cost, nontoxicity, chemical stability, and a highly efficient charge transfer process [26]. In a KOH electrolyte, one atomic percentage of Ni-doped SnO_2_ exhibits a specific capacitance of 793 F/g and a current density of 2.5 A/g. This capacitance is preserved for 1250 cycles [25]. Meng et al. synthesized 3D Ni/SnO_2_ nanoflowers hydrothermally, and the resultant material had a specific capacitance of 410 mF cm^−2^ at a current density of 1 mA cm^−2^ in a 1M NaOH electrolyte [27].

The methods reported for preparing mixed semiconductor oxide nanostructures encompass colloidal growth, hydrothermal synthesis, microwave combustion, the sol–gel process, chemical vapor condensation, spray pyrolysis, solution casting, sputtering, and laser ablation in liquids and gases [28,29,30,31,32,33,34,35]. Moreover, the practicability of electrode materials containing carbon, metal oxides, metal sulfides, conducting polymers, etc., and their composites needs extensive research, making the investigation of efficient new electrode materials superfacial. Interestingly, the energy density of a supercapacitor device is determined by the electrode and the electrolyte component present in it. Therefore, the selection of electrolytes greatly affects the supercapacitor performance [14]. Among different electrolytes, aqueous electrolyte is the most widely adopted in the literature owing to its superior ionic conductivity and exceptional safety properties. However, its narrow voltage window is its most significant drawback [36]. To solve this aforementioned problem and to attain enhancement of the capacitance or energy density of the supercapacitor, a new concept known as water-in-salt is proposed, which involves adding a minor amount of a redox additive/mediator into the electrolyte, which can increase the potential window [37]. With the addition of a K_3_[Fe(CN)_6_]/K_4_[Fe(CN)_6_/hydroquinone redox additive into a common electrolyte such as Na_2_SO_4_, KOH, KI, H_2_SO_4_, etc., the specific capacitance of the supercapacitor can be increased by several times that of its performance in a common electrolyte [38]. Generally, common electrolytes are electrochemically inert with no redox reactions occurring in them [39]. Whereas, in redox-additive-based electrolytes, the redox reactions take place at both the electrode surface and electrolyte, which leads to the enhancement of the supercapacitor’s performance. Inspired by the abovementioned concerns, in this work, novel Ni-doped SnO_2_ with a microplate-type morphology has been synthesized using a template-free hydrothermal technique. It is simple, affordable, and nontoxic [35]. In addition, the precursor’s effect on the structure, morphology, and electrochemical performance in a 6 M KOH electrolyte and a 6 M KOH + 0.1 M K_4_[Fe(CN)_6_]·3H_2_O redox additive electrolyte (RAE) was discussed.

## 2. Experimental Procedure

### 2.1. Synthesis of Ni_1−x_Sn_x_O_2_ (x = 0, 0.001, 0.003, 0.005) Microplates

The hydrothermal method was used to prepare pristine and Ni-doped SnO_2_ nanostructures. In the initial step, the stoichiometric amount of tin (IV) chloride pentahydrate (SnCl_4_·5H_2_O) (0.009 M, Sigma Aldrich AR grade, St. Louis, MO, USA) and nickel (II) chloride hexahydrate (0.001 M Sigma Aldrich AR grade) precursors were dissolved in 100 mL double distilled water. The mix was then subjected to extensive stirring to obtain a stannous solution. Then, a few drops of sodium hydroxide (NaOH) solution were mixed into the above solution to maintain a pH of ∼8.0, and stirring was resumed to form a homogeneous solution. The resultant suspension was then sealed in a Teflon-lined stainless steel autoclave and kept inside a muffle furnace, maintained at 180 °C/12 h. After the hydrothermal treatment, it was cooled to room temperature naturally, and the precipitate was collected by centrifugation with excess distilled water and ethanol, and thoroughly dried under vacuum at 80 °C/12 h. Subsequently, the product was subjected to calcination at 600 °C maintained for 4 h with a heating rate of 1°/min. to obtain the final product, referred to as 1% Ni-doped SnO_2_ nanocrystals with the formula Ni_1−x_Sn_x_O_2_ (x = 0.001). The prepared nanoparticles NPs had no impurities. A similar procedure was adopted for the synthesis of pristine and Ni_1−x_Sn_x_O_2_ (x = 0.003, 0.005) nanostructures. The reaction process occurring during the synthesis of pristine SnO_2_ was as follows [40]:

$${\mathrm{Sn}}^{4+}+4{\mathrm{OH}}^{-}\;\to \;\mathrm{Sn}{\left(\mathrm{OH}\right)}_{4}↓$$

$$\mathrm{NaOH}\;\to {\mathrm{Na}}^{+}+{\;\mathrm{OH}}^{-}$$

$${\mathrm{SnCl}}_{2}+2\mathrm{NaOH}\;\to \mathrm{Sn}{\left(\mathrm{OH}\right)}_{2}↓+2\mathrm{NaCl}$$

$$\mathrm{Sn}{\left(\mathrm{OH}\right)}_{2}↓/\mathrm{Sn}{\left(\mathrm{OH}\right)}_{4}↓\;\overset{\Delta }{\Rightarrow }{\;\mathrm{SnO}}_{2}+2H_{2}O$$

**Figure 1:**
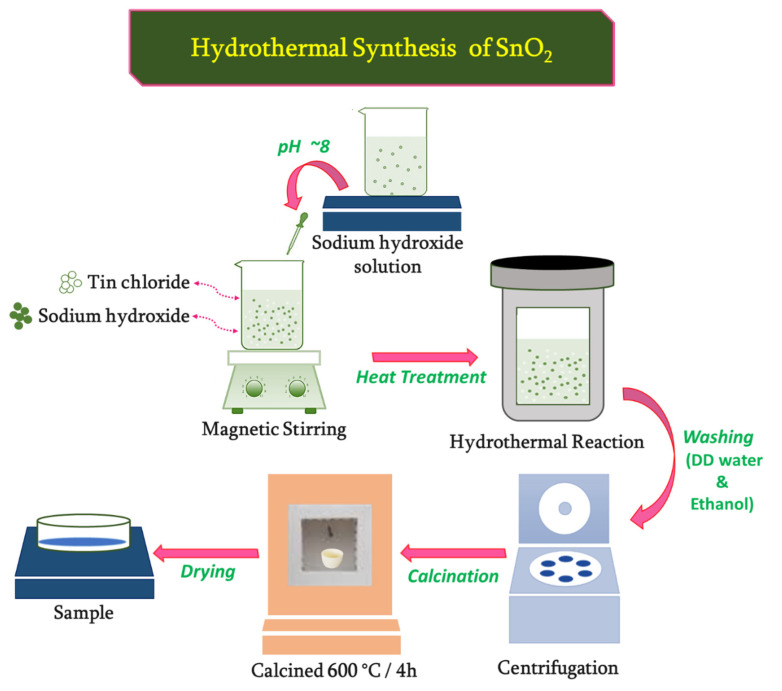
Hydrothermal synthesis of SnO_2_ composite.

The steps involved in the synthesis process of SnO_2_ by the hydrothermal method is displayed in Figure 1.

### 2.2. Characterization Techniques of Synthesized Nanostructures

The phase purity and crystal structure of the final products were explored using a Bruker D8 advanced ECO X-ray diffractometer in the 2(θ) range of 10 to 80°. A scanning electron microscope (SEM; ZEISS EVO 18, ZEISS, Jena, Germany) connected to an energy-dispersive X-ray spectrometer (EDS-Bruker-X Flash 6130, Bruker, Billerica, MA, USA) was utilized to assess the surface morphology and chemical elemental percentage. The Shimadzu IR Tracer 100 spectrometer was used to record the Fourier transform infrared (FTIR) spectra at the wavenumbers 400 to 4000 cm^−1^. The electrochemical analyses of the calcined samples were performed using CH-Instrument Model 6008e (CH Instruments, Inc., Bee Cave, TX, USA).

### 2.3. Preparation of Electrode for Supercapacitor Investigations

The supercapacitor studies of modified working electrodes were performed through cyclic voltammetry (CV), galvanostatic charge discharge (GCD), and electrochemical impedance spectroscopy (EIS), in a standard three-electrode configuration utilizing a platinum (Pt) (counter electrode) and Ag/AgCl electrode (reference electrode). The working electrode was prepared from different samples and analyzed with a 6 M potassium hydroxide (KOH) electrolyte solution. The electrolyte plays a key role in improving the C_sp_ of the material and the redox additive electrolyte (RAE) exhibits an increased electrochemical performance [23]. Therefore, 6 M KOH + 0.1 M K_4_[Fe(CN)_6_]·3H_2_O as the RAE was utilized and its electrochemical performance was tested. The doctor blade technique was used for the fabrication of the modified working electrode. The working electrode was fabricated by utilizing 85 wt% of the active prepared sample, 10 wt% of active carbon, and 5 wt% of polyvinylidene difluoride (PVDF). Until homogeneity, the aforementioned materials were triturated in the presence of N-methyl pyrrolidinone (NMP). The mix was pasted on a Nickel foil, which was later dried under vacuum at 60 °C/12 h. Prior to this, a 1 cm^2^ square of thin Ni foil was cleansed by treating it consecutively with acetone, 1 M hydrochloric acid, ethanol, and DD water.

## 3. Results and Discussion

### 3.1. XRD Analysis

**Figure 2:**
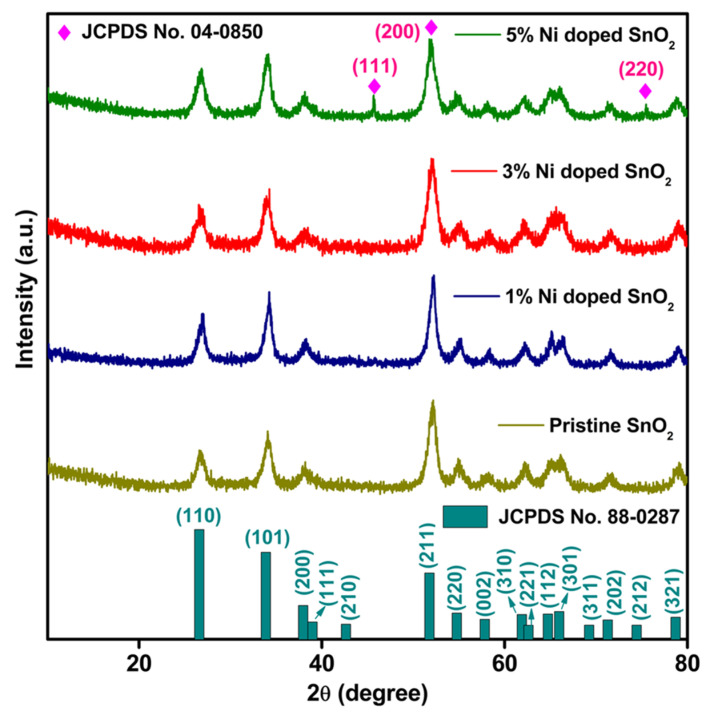
XRD pattern of pristine and Ni-doped SnO_2_ composites.

The XRD patterns of the pristine SnO_2_ and Ni/SnO_2_ composites are presented in Figure 2. All the experimental diffraction patterns display sharp and well-defined peaks, signifying the crystallinity of the final prepared materials. The detected major 2θ values of all the patterns exhibit the tetragonal structure, $P4_{2}/mnm$ *(136)* space group, and lattice parameters a = b = 3.802 Å and c = 4.836 Å of SnO_2_ and are in agreement with the standard JCPDS # 88-0287 data. The observed peaks at 26.59° (110), 33.94° (101), 38.18° (200), 39.12° (111), 51.87° (211), 54.86° (220), 57.98° (002), 62.53° (221), 65.03° (110), 66.11° (301), 71.28° (202), 74.52° (212), and 78.89° (321) are the characteristic 2θ values and their (hkl) respective planes of SnO_2_. When Ni is introduced as the dopant in the host SnO_2_, all the diffraction peaks of SnO_2_ with additional diffraction peaks of Ni showing a mixed phase can be identified. The secondary phases identified for the Ni-doped SnO_2_ composites at 2θ = 44.56° (111), 51.74° (200), and 75.71° (220) are indexed to the standard JCPDS # 04-0850 data, indicating Ni has a cubic structure with an *Fm*$\bar{3}$*m (225)* space group. Moreover, some of the diffraction peaks of SnO_2_ are shifted towards a lower Bragg 2θ angle. This is because of the ionic radius of Ni^2+^ (0.69 Å) being lesser than that of Sn^2+^ (1.18 Å) [33,41]. The average crystallite size (D) is estimated using the Scherrer equation.

$$D=\frac{K\lambda }{\beta cos\theta }$$

where *K*—constant, *λ*—1.5405 Å, *β*—FWHM, and *θ*—Bragg diffraction angle. The calculated average crystallite sizes are 22.19, 24.99, 21.06, and 13.82 nm for SnO_2_ and Ni (1, 3 and 5%)-doped SnO_2_, respectively. Furthermore, the broad peaks detected in the diffraction pattern are due to the smaller crystallite size, and along with the sharp peaks expose the crystalline nature of the sample, as an effect of the Ni dopant [40].

### 3.2. FTIR

**Figure 3:**
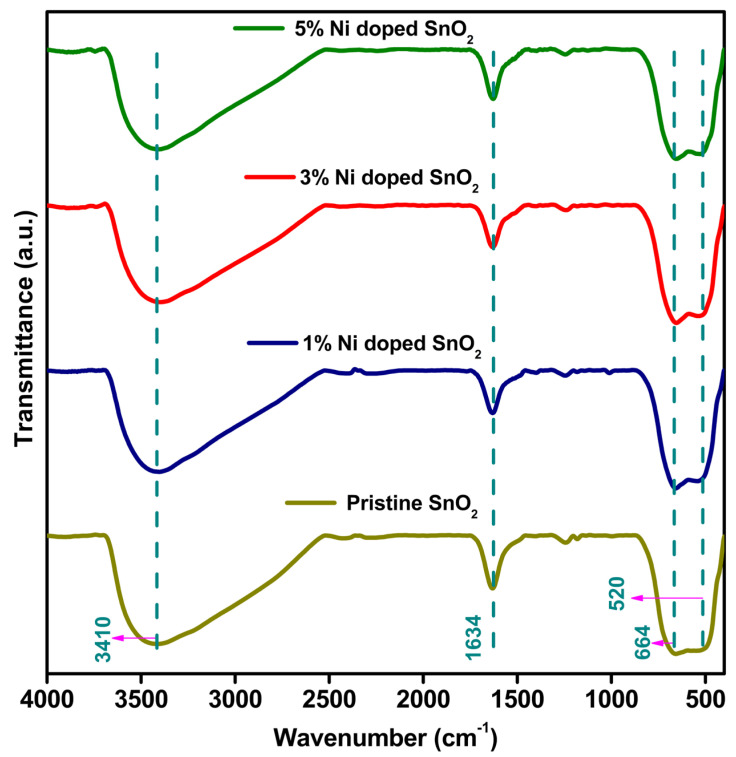
FTIR spectra of pristine and Ni-doped SnO_2_ composites.

Figure 3 presents the FTIR spectra of the pristine and Ni-doped SnO_2_ nanocomposites. The peak observed for all the samples at approximately 610–930 cm^−1^ represents the stretching modes of Sn–O–Sn [42]. The absorption band near 520 and 664 cm^−1^ is connected to the antisymmetric Sn–O–Sn stretching mode on the surface linking oxide produced by the condensation of the neighboring surface OH groups [40]. A broad intensity band appearing at 3410 cm^−1^ and an absorption peak at 1634 cm^−1^ were identified for all the samples, representing the O-H stretching vibration and H_2_O functional groups [33,40]. There were no additional peaks detected in the pristine and Ni-doped SnO_2_ composites, indicating the purity of the sample.

### 3.3. SEM, EDS, and Mapping

**Figure 4:**
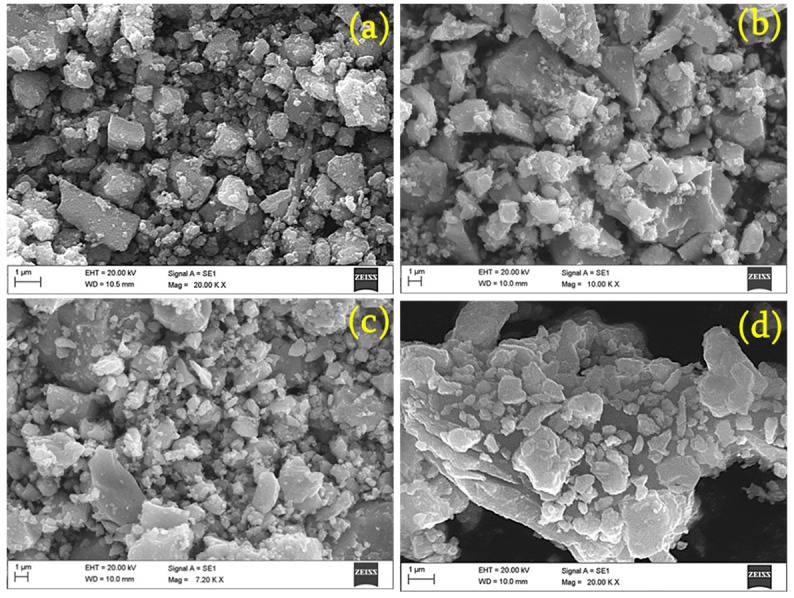
Micro-scale SEM image of (**a**) pristine, (**b**) 1% Ni- (**c**) 3% Ni-, and (**d**) 5% Ni-doped SnO_2_ composites.

**Figure 5:**
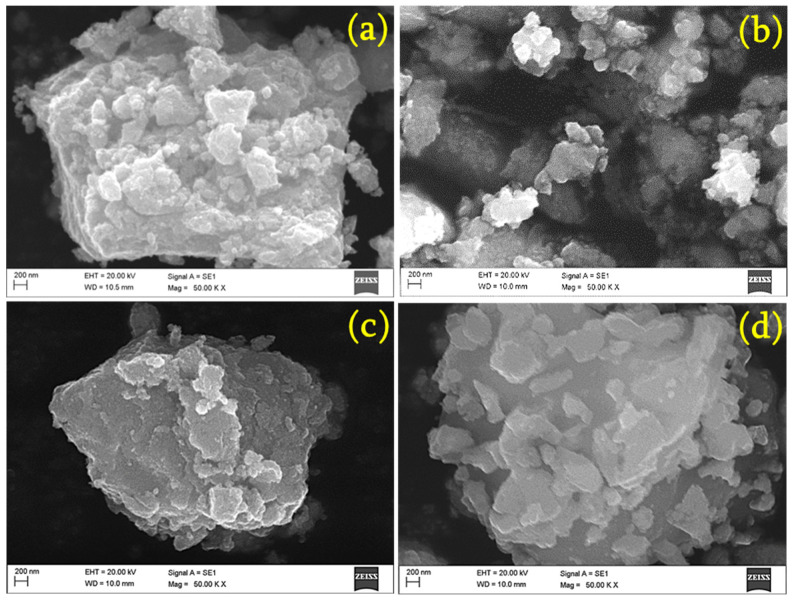
Nano-scale SEM image of (**a**) pristine, (**b**) 1% Ni- (**c**) 3% Ni-, and (**d**) 5% Ni-doped SnO_2_ composites.

The different magnified SEM images of the samples were investigated to understand their morphological features. Figure 4a–d display micro-scale images of the pristine and Ni (1, 3, and 5%)-doped SnO_2_, respectively. An irregular flake-like structure is clearly observed for all the samples. Figure 5a–d show a nano-scale image of the pristine and Ni (1, 3, and 5%)-doped SnO_2_, respectively. The images clearly show a large number of homogeneously sized particles. The majority of the particles are on the nano scale. Nuclei of crystals aggregate together to form these particles, which form nanoclusters, and then undergo assembly, unification, and preferential growth into micro/nanoflakes [43]. The dopant Ni has no major impact on the morphology [33,44,45].

**Figure 6:**
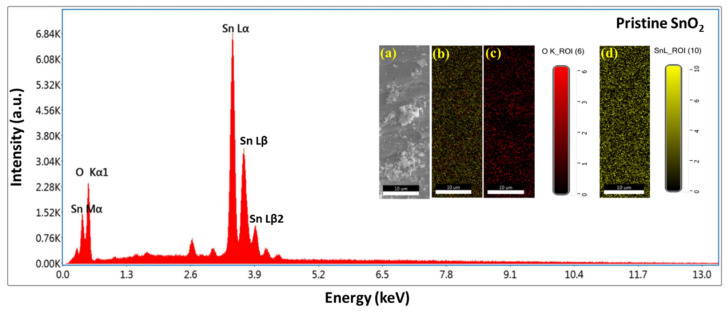
EDS spectrum of pristine SnO_2_ composites; inset shows elemental mapping images: (**a**) scan area SEM image, (**b**) element distribution in composite, individual element scan of (**c**) O and (**d**) Sn.

**Figure 7:**
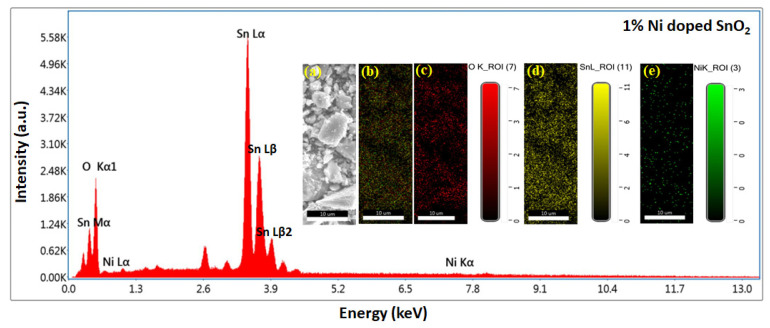
EDS spectrum of 1% Ni-doped SnO_2_ composites; inset shows elemental mapping images: (**a**) scan area SEM image, (**b**) element distribution in composite, individual element scan of (**c**) O, (**d**) Sn and (**e**) Ni.

**Figure 8:**
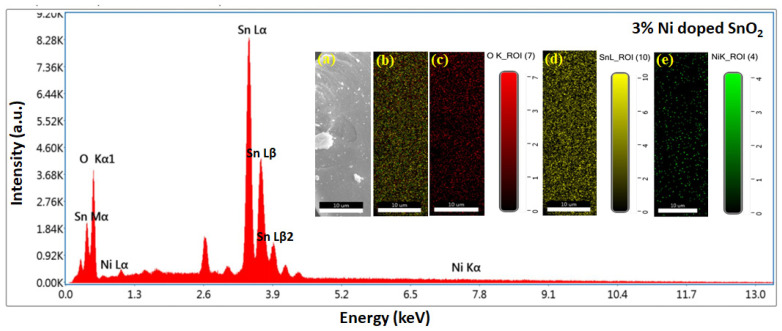
EDS spectrum of 3% Ni-doped SnO_2_ composites; inset shows elemental mapping images: (**a**) scan area SEM image, (**b**) element distribution in composite, individual element scan of (**c**) O, (**d**) Sn and (**e**) Ni.

**Figure 9:**
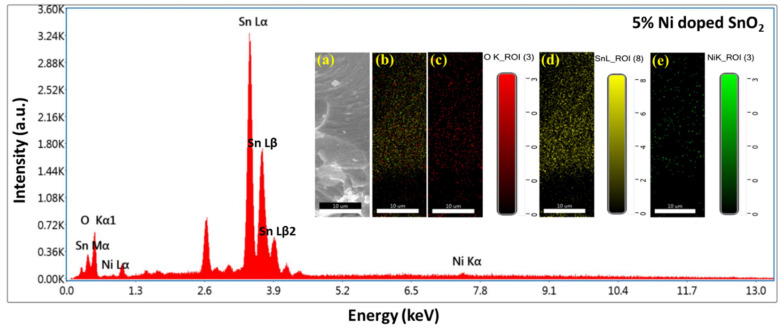
EDS spectrum of 5% Ni-doped SnO_2_ composites; inset shows elemental mapping images: (**a**) scan area SEM image, (**b**) element distribution in composite, individual element scan of (**c**) O, (**d**) Sn and (**e**) Ni.

The EDS and mapping results are displayed in Figure 6, Figure 7, Figure 8 and Figure 9. Oxygen and tin were present in the pristine SnO_2_ (Figure 6) and oxygen, tin, and nickel were present in the Ni (1, 3, and 5%)-doped SnO_2_ samples (Figure 7, Figure 8 and Figure 9), which confirms the presence and formation of SnO_2_ and Ni structures. The atomic ratios of Sn, O, and Ni in the composites agree well with the chemical composition of Ni/SnO_2_. To determine the existence and distribution of Ni in the SnO_2_ host structure, the elemental mapping of Ni/SnO_2_ was also performed for all the samples (shown in inset). The areas of bright contrast correlate with the O, Sn, and Ni signals, with the map showing the element distribution in the composite (Figure 6a,b, Figure 7a,b, Figure 8a,b and Figure 9a,b). The individual element distribution is also shown (Figure 6c,d, Figure 7c–e, Figure 8c–e and Figure 9c–e). The EDS and mapping results of all the samples exhibit clear signals of Sn, Ni, and O, and no additional peaks, indicating the existence of impurities was not detected, indicating that the prepared nanomaterials that were generated from the starting precursors were impurity-free. Consequently, the structural and elemental analyses results provide strong evidence of the Ni dopant in the SnO_2_ composite.

### 3.4. Electrochemical Studies

The electrochemical supercapacitive behavior of the working active electrode was studied at room temperature using the CV, GCD, and EIS techniques.

#### 3.4.1. Cyclic Voltammetry Analysis

**Figure 10:**
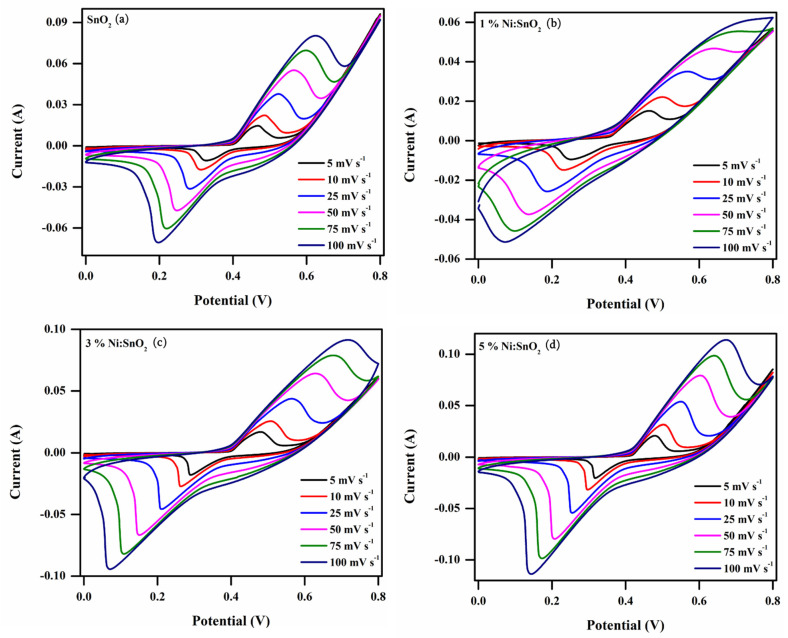
CV curves of (**a**) pristine and (**b**–**d**) Ni-doped SnO_2_ nanostructures at various scanning rates (5–100 mVs^−1^).

The fabricated electrodes were analyzed by CV at various scanning rates of 5 to 100 mVs^−1^ in the potential range of 0 to 0.8 V, as presented in Figure 9. The CV profile illustrates a non-rectangular profile, signifying the faradaic redox reactions representing the pseudocapacitance profile. There were no distinct redox peaks detected at the various scan rates, suggesting that the electrodes were charged and discharged at a pseudoconstant rate over the complete voltammetric cycle [23]. The current densities of the oxidation and reduction peaks are raised when the scan rate is raised, which may be caused by quasi-reversible reactions that happen at the electrode and electrolyte interface [33]. When the scan rate increases, a peak shift is observed in the oxidation and reduction peaks. In an aqueous KOH electrolyte, the faradic reactions of the SnO_2_ electrode’s electrochemical reaction may be as follows:

$${\mathrm{SnO}}_{2}+{\;\mathrm{xK}}^{+}+{\;\mathrm{xOH}}^{-}\;\to {\mathrm{KxSnO}}_{2}$$

$${\mathrm{SnO}}_{2}+{\;H}_{2}O+e^{-}\;↔\mathrm{SnOOH}+{\;\mathrm{OH}}^{-}$$

$$\mathrm{SnOOH}+{\;e}^{-}\;↔\mathrm{SnO}+{\;\mathrm{OH}}^{-}$$

where x is the mole fraction of the involved K^+^ ions during the reaction. The specific capacitance (*C_sp_*) (F g^−1^) is calculated using the following equation and is tabulated in Table 1:

$$C_{sp\left(CV\right)}=\frac{\int I\;dV}{Sm\Delta V}$$

where ∫I dV—integral area of the CV curve, *S*—scanning rate (mV s^−1^), *m*—utilized sample mass (mg), and Δ*V*—potential window (V). A higher area under the curve and higher current response value is detected for the 5% Ni-doped SnO_5_ sample, resulting in a rise in capacitance. A histogram displaying the scan rate and its respective *C_sp_* value for all the sample is shown in Figure 10. The maximum *C_sp_* value observed is 841.85 Fg^−1^ at 5 mV s^−1^ for 5% Ni/SnO_2_. When the scanning rates were increasingly raised, the electrolyte ions would not have had sufficient time to reach the electrode material surface completely, which may be the cause for the decreasing C_sp_ value. At greater scanning rates, only partial redox reactions at the electrode surface may occur and are unfavorable for electrolyte adsorption [46].

**Figure 11:**
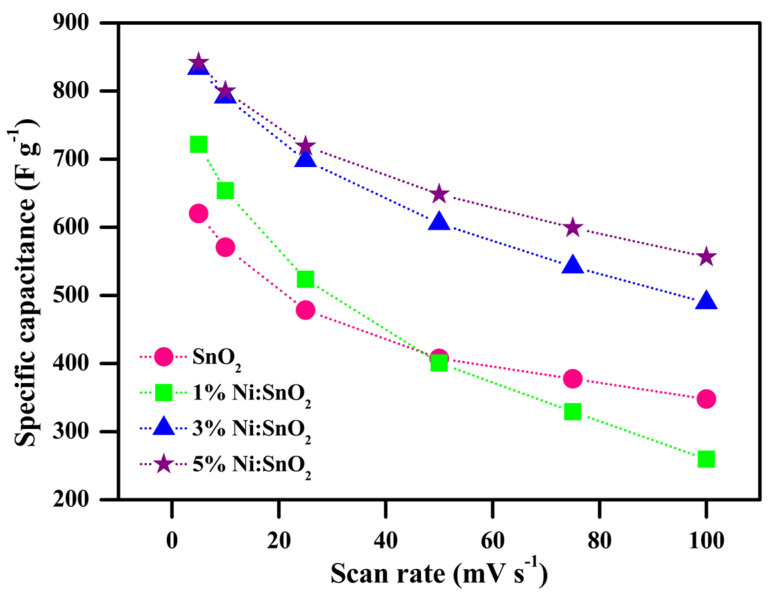
C_sp (CV)_ versus scanning rate of pristine and Ni-doped SnO_2_ nanocomposites.

Figure 11 shows the C_sp_ trend corresponding to the scanning rate of all samples.

**Figure 12:**
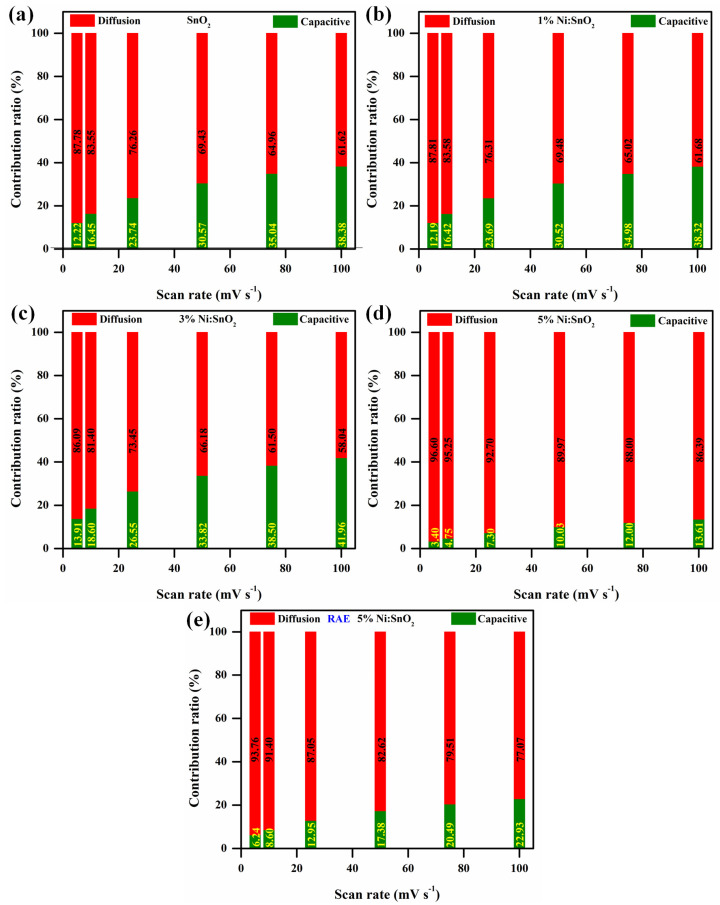
Comparison of capacitive contribution and diffusion-controlled contribution of (**a**) SnO_2_, (**b**–**d**) X% Ni/SnO_2_, and (**e**) 5% Ni/SnO_2_ using RAE.

**Figure 13:**
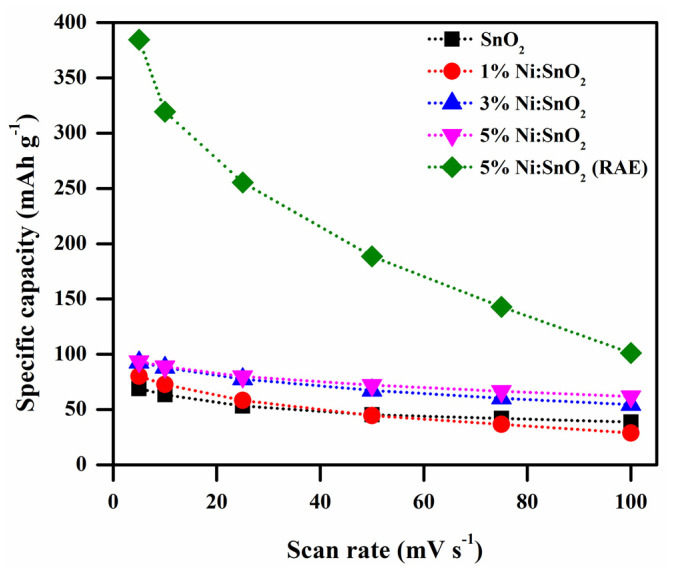
Specific capacity versus scan rate.

The total capacitance of an electrode encompasses two components: 1. the fast electrochemical reaction caused by ion adsorption/desorption (i.e., EDLC process), and 2. the fast faradaic reaction of redox species caused by the ion diffusion between the electrode—electrolyte material. It is essential to know the dominating process involved in the electrochemical reaction to better understand the performance of the electrode material. The following relationship determines an electrode’s current during a linear scan with a constant scan rate [47,48].

$$i=a\nu^{b}$$

where *a* and *b* are constants, *ν* is the scan rate, and *i* is the current. In this case, the electrode material’s two distinct responses are explained by the value of the “*b*” (0.5 or 1). Whereas the charge storage mechanism for *b* = 1 is primarily non-faradaic and capacitive or non-diffusive and controlled, as is frequently observed for EDLC electrode systems, for *b* = 0.5, the charge storage mechanism is dominated by the faradic adsorption/desorption of ions from electrolyte to electrode, i.e., a diffusion-controlled process typical for battery-type electrode materials [49]. For the pristine nanocomposites, X% Ni/SnO_2_ (X = 1, 3, and 5), and RAE, the computed b values are 0.5643, 0.5711, 0.5717, 0.5757, and 0.5722, respectively. By considering the total CV current (i) as the sum of the diffusion-controlled and capacitive currents as well as the adsorption/desorption (diffusion-controlled and combined currents), pseudocapacitive and battery materials may frequently be identified from their quantitative kinetics utilizing a power law [49,50] we may divide the equation as follows:

$$i\left(V\right)=k_{1}\nu +k_{2}\nu^{1/2}$$

where the parameters *k*_1_(*ν*) and *k*_2_(*ν*^1/2^) represent the adsorption/desorption current and capacitive current, respectively. We may obtain the slope *k* by simply charting the connection between *i* and *ν*^0.5^. This allows us to depict the capacitive and diffusive contributions as seen in Figure 12 by computing the *k* value at different voltages at varied scan speeds. The diffusive contribution is predominant in the sample, as seen in the Figure 12. Therefore, this relation is used to compute the specific capacity:

$$C_{s}=\frac{\int I\;dV}{S\times m\times 3.6}$$

where *C_s_* is the specific capacity (mAh g^−1^), ∫I dV is the area of the CV profiles, *S* is the scan rate, and *m* is the mass of the active material. The maximum specific capacities observed in 6 M KOH electrolyte are 68.92, 80.19, 92.61, and 93.62 mAh g^−1^ for SnO_2_, 1% Ni/SnO_2_, 3% Ni/SnO_2_, and 5% Ni/SnO_2_, at a scan rate of 5 mV s^−1^, respectively. The superior *C_s_* observed is 384.64 mAh g^−1^ (scan rate of 5 mV s^−1^) for 5% Ni/SnO_2_ using a redox additive electrolyte. Figure 13 shows the specific capacity versus the scan rate.

#### 3.4.2. Galvanostatic Charge–Discharge Analysis

**Figure 14:**
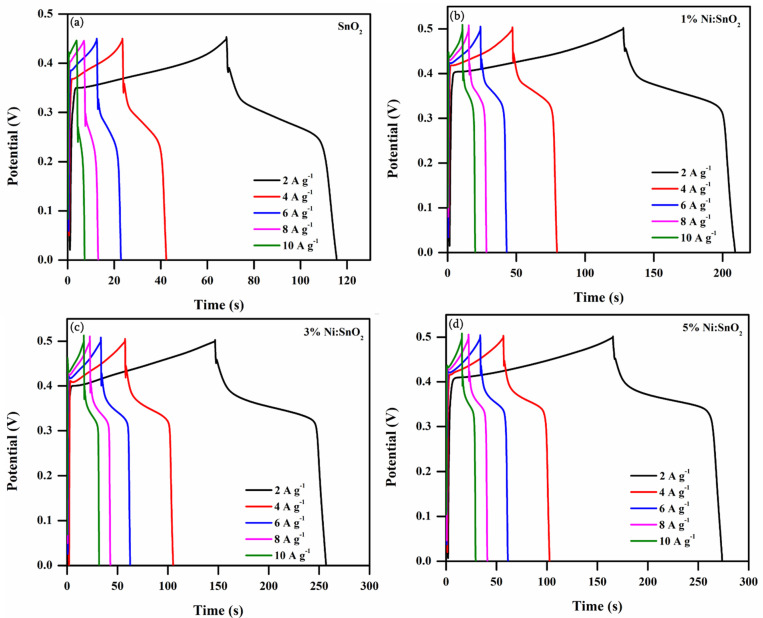
GCD profile of (**a**) pristine and (**b**–**d**) Ni-doped SnO_2_ nanocomposites at different current densities (2 to 10 Ag^−1^).

GCD analysis is a reliable technique for assessing the electrochemical performance of electrode materials with controlled current conditions. Figure 14a,d illustrate the GCD profiles of four different samples attained with the potentials 0 to 0.5 V at different current densities from 2 to 10 A g^−1^, employed using a 6 M KOH electrolyte. The non-linearity of the discharging curves displayed in all the samples’ GCD profiles suggests the involvement of pseudocapacitance owing to the faradaic reaction [43]. Nearly identical charge–discharge curves are presented for all the current densities representing reversible faradaic electrochemical reactions, which also suggests a reliable charge–discharge Coulombic capability, meaning there is less electrode polarization in the pseudocapacitive profile [44,51].

**Figure 15:**
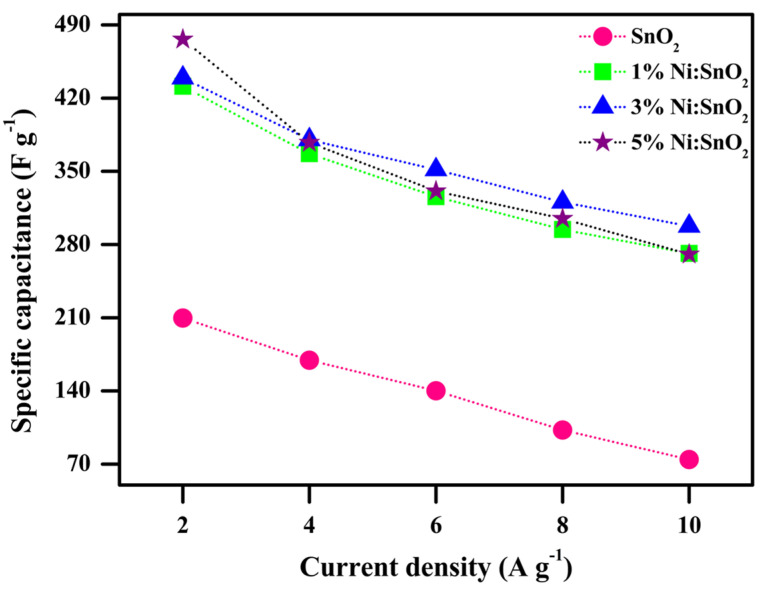
C_sp(GCD)_ vs. current density of pristine and Ni-doped SnO_2_ samples.

**Figure 16:**
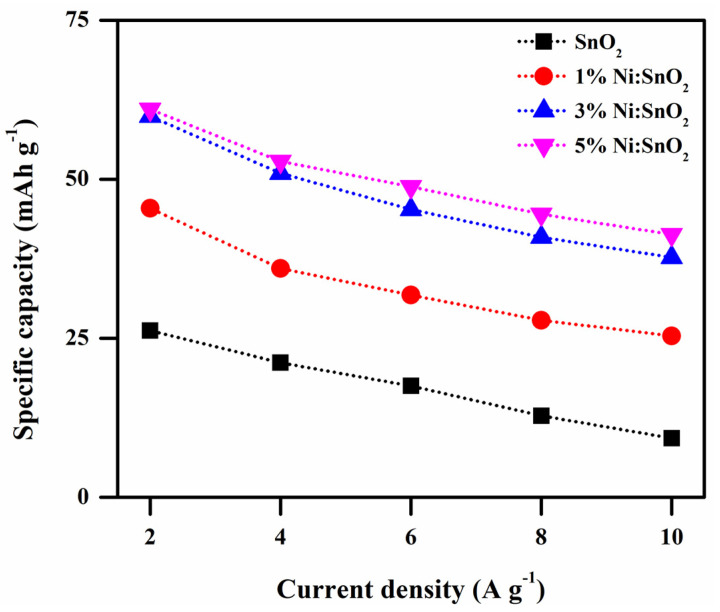
Specific capacity vs. current density of pristine and Ni-doped SnO_2_.

The *C_sp_* (F g^−1^) and *C_s_* (mAh g^−1^), values from the *GCD* curves are measured and shown in Table 2 using

$$C_{sp\left(GCD\right)}=\frac{Idt}{mdV}$$

$$C_{s\left(GCD\right)}=\frac{Idt}{m\times 3.6}$$

where *I*—current density (A g^−1^), *dt*—discharge time (s), *m*—mass of the active sample (g), and *dV*—applied potential (V). *C_sp_* versus current density calculated for all four samples is presented in Figure 15. A higher *C_sp_* value is obtained at a lower current density. At a lower current density, the ions diffuse from the electrolyte and are almost fully accumulated at the charge carriers in all the existing inner active sites of the electrodes. The higher *C_sp_* value attained is 476.22 F g^−1^ for the 5% Ni-doped SnO_2_ nanocomposite at 2 A g^−1^ current density. In contrast, electrode and electrolyte ion active interaction is significantly minimized at higher current densities, resulting in a decrease in *C_sp_* [52,53]. The superior *C_sp_* for the 5% Ni-doped SnO_2_ may be because of its smaller crystallite size, morphology, slightly increased active sites, and large surface area compared to the other three samples, which manifestly boosts the active sites’ involvement in the diffusion of ions, thereby permitting electronic and ionic transport [52]. From the CV and GCD results, the Ni-doped SnO_2_ sample exhibits a much better *C_sp_* than that of the pristine sample in the same applied conditions. The specific capacities were calculated for the prepared electrode materials and the calculated values are 26.21, 45.44, 59.91, and 61.01 mAh g^−1^ for the pure 1%, 3%, and 5% Ni-doped SnO_2_ nanocomposite at 2 A g^−1^ current density. Figure 16 shows the specific capacity versus the current density of the electrode material.

#### 3.4.3. Electrochemical Impedance Spectroscopy

**Figure 17:**
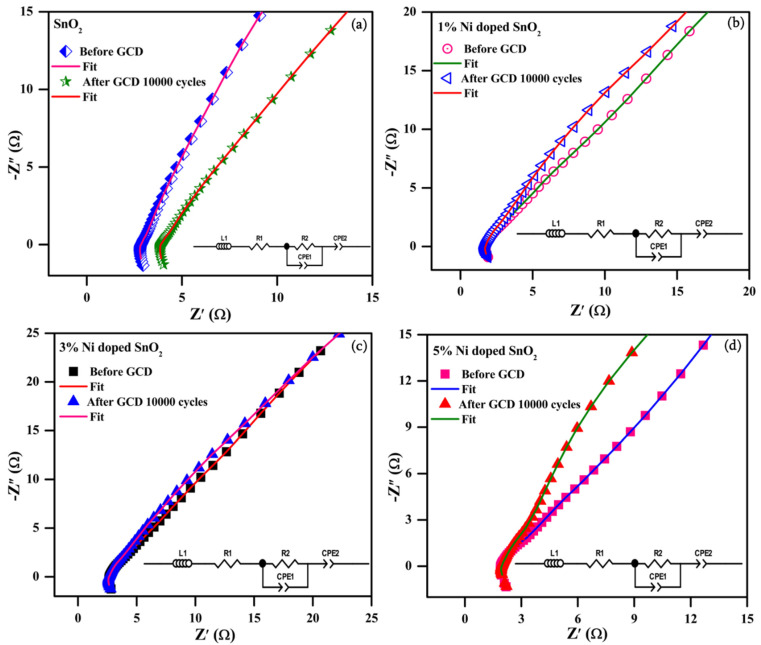
EIS spectra for (**a**) SnO_2_; (**b**) 1% Ni-doped SnO_2_; (**c**) 3 % Ni-doped SnO_2_; (**d**) 5% Ni-doped SnO_2_ and prior to the 10,000 GCD cycles in 6 M KOH electrolyte.

The EIS spectral analysis data for the modified active electrode previous and prior after the 10,000 GCD cycles in the 6 M KOH electrolyte were recorded and displayed in Figure 17a–d (equivalent circuit insert) with data fitting (Z view 4.0 software used). Typical Nyquist plots for all the samples were estimated at the 1 Hz and 100 kHz frequency with a potential amplitude of 5 mV. All the samples reveal electrochemical reactions of adsorbed charge carriers on the electrode surface interface with electrolytes, signified by high-frequency inductance, and are displayed in Figure 14a–d.

The Nyquist spectra are identical, without much variation in shape, for all the electrode samples both previous and after the 10,000 GCD cycles, proving the stability of the electrodes. A semicircle in the high-frequency (HF) portion and an approximate linear component in the low-frequency (LF) portion with an identical gradient to the imaginary axis suggests rapid kinetics diffusion progression with low resistance [44,54]. The Nyquist plot in the higher-frequency region is noted to be an imperfect semicircle. This imperfect semicircle is caused by the uneven current distribution on the surface of the Ni/SnO_2_ electrode. The calculated R_s_ values for the SnO_2_ and Ni (1, 3, and 5%)-doped SnO_2_ samples are as follows: 2.82, 1.72, 2.64, and 1.89 Ω for before the GCD cycles, and 3.87, 1.76, 2.68, and 1.97 Ω for after the 10,000 GCD cycles, respectively. The corresponding R_ct_ values are as follows: 1.02, 1.28, 0.61, and 2.19 Ω for before the GCD cycles, and 1.05, 1.30, 0.79, and 2.31 Ω for after the 10,000 GCD cycles. The calculated R_s_ and R_ct_ values for all the electrodes are raised after the 10,000 GCD cycles, which might be because of the limited decay of the electrode conductivity during the charge–discharge process [44]. The R_s_ value decreased and the R_ct_ value increased as an impact of the Ni dopant in SnO_2_. Furthermore, in all the electrochemical techniques, the 5% Ni-doped SnO_2_ exhibited a better electrochemical performance. These characteristics might be due to the synergistic effects and the ideal combination of the components, leading to a better supercapacitive performance [55,56,57].

#### 3.4.4. Electrochemical Performance of 5% Ni-Doped SnO_2_ in Additive Electrolyte

**Figure 18:**
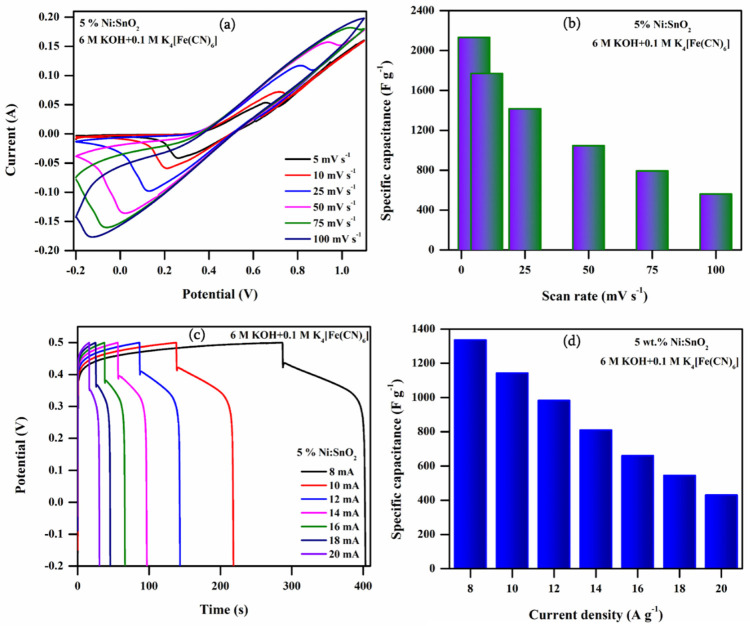
(**a**) CV profiles at different scanning rates, (**b**) Csp (CV) vs. scan rate, (**c**) GCD profile at different current densities, and (**d**) Csp(GCD) versus current density of 5% Ni-doped SnO_2_ in RAE.

**Figure 20:**
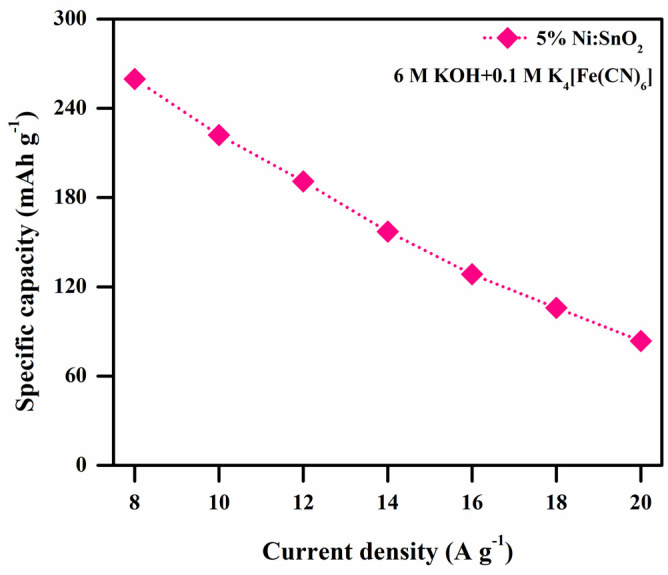
Specific capacity versus current density of 5% Ni-doped SnO_2_ in RAE.

Redox additives instantly dissolve in electrolytes, highlighting this approach as safer and easy to scale up. It is possible to attribute the improved electrochemical performance stimulated by the addition of RAE to it readily enhancing the ionic conductivity and also generating additional redox pairs to take part in redox reactions [30]. Hence, to further improve the electrochemical performance, 6 M KOH + 0.1 M (K_4_[Fe (CN)_6_]·3H_2_O was used as the RAE and was investigated with the 5% Ni-doped SnO_2_ sample. The CV and GCD curves at different scanning rates and current densities are presented in Figure 18a–d. The C_sp_ values are calculated from the CV (shown in Table 1) and GCD curves. The calculated C_sp_ values from the GCD curves are 1335.77, 1141.85, 981.92, 808.41, 660.34, 544.80, and 430.01 F g^−1^ at current densities of 8, 10, 12, 14, 16, 18, and 20 A g^−1^, respectively. The maximum C_sp_ value obtained is 2130.33 F g^−1^ at 5 mV s^−1^ according to the CV analysis and 1335.77 F g^−1^ (maximum specific capacity of 259.73 mAh g^−1^; shown in the Figure 20) at 8 A g^−1^ according to the GCD analysis.

**Figure 19:**
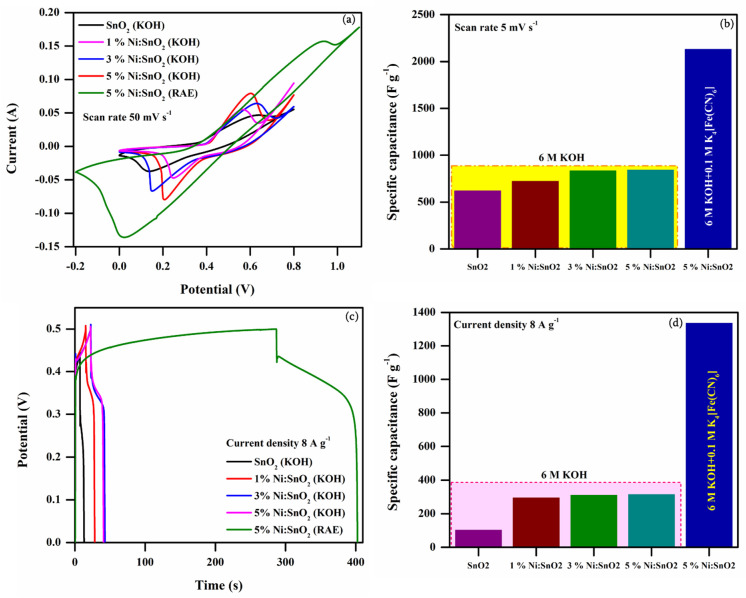
Comparison of (**a**) CV profile, (**b**) C_sp(CV)_ vs. scanning rate at 5 mV s^−1^, (**c**) comparison of GCD profile, and (**d**) C_sp(GCD)_ vs. current density at 8 A g^−1^.

Figure 19a–d present the CV and GCD profiles of four different samples obtained at the same scanning rate of 50 mV s^−1^ and current density of 8 A g^−1^, respectively, in KOH and 6 M KOH + 0.1 M (K_4_[Fe (CN)_6_]·3H_2_O as the RAE. The dramatic change in the integral area under the CV curve and the longer discharge time are observed for the sample that underwent electrochemical studies in the RAE. The C_sp_ value is approximately 2.5- to 3.4-fold higher than the electrochemical performance of the sample that underwent study in the KOH electrolyte.

**Figure 21:**
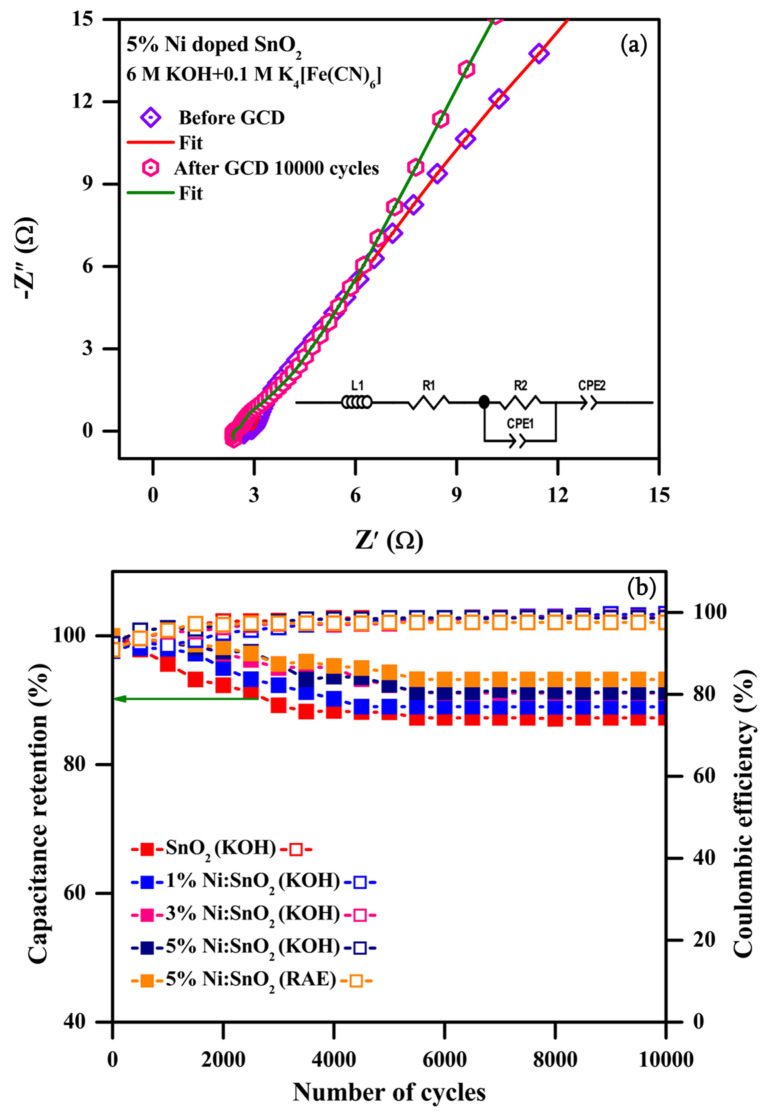
(**a**) Nyquist pot of 5% Ni/SnO_2_ in RAE and (**b**) cyclic stability and Coulombic efficiency after 10,000 cycles for all samples in KOH electrolyte and 5% Ni-doped SnO_2_ in RAE.

Figure 20 shows the plot of specific capacity versus current density of 5% Ni-doped SnO_2_ in the redox additive electrolytes. The maximum specific capacitance value obtained at the low current density is 8 A g^−1^. Figure 21a shows the Nyquist plot of the 5% Ni-doped SnO_2_ studied in the RAE. The EIS spectra are similar to those with the KOH electrolyte. The estimated R_s_ values are 2.42 and 2.73 Ω and the R_ct_ values are 0.18 and 0.21 Ω, respectively, for before and after the 10,000 GCD cycles. The R_ct_ values are much lower than the values obtained in the KOH electrolyte. The redox additives in the aqueous electrolyte greatly boost the electrochemical performance because of their capability to enhance the charge storage capacity via redox transformation. The C_sp_ of the supercapacitor was boosted as a result of intercalating and accumulating redox species due to reversible redox reactions at the electrode/electrolyte interface as well as on the electrode’s surface. The reversible redox reaction of the redox additive is given as follows [30]:

$${\left[\mathrm{Fe}{\left(\mathrm{CN}\right)}_{6}\right]}^{3-}+e^{-}\;↔\;{\left[\mathrm{Fe}{\left(\mathrm{CN}\right)}_{6}\right]}^{4-}$$

The KOH electrolyte provides K^+^ and OH^-^ ions for employment of electrode and additional capacitance from the reversible [Fe(CN)_6_]^3−^/[Fe(CN)_6_]^4−^ [30,37].

Prolonging the cycling performance is a vital factor in defining the practical applications of electrodes for supercapacitors. In Figure 21b, the long cycling stability tests for the 10,000 continuous GCD cycles and the corresponding Coulombic efficiency values are presented at 8 A g^−1^ for the pristine SnO_2_, 1% Ni/SnO_2_, and 3% Ni/SnO_2_ samples in the KOH electrolyte and for the 5% Ni/SnO_2_ sample both in the KOH electrolyte and RAE. All the samples exhibit excellent stability, with the retention of 87.26, 88.99, 91.12, 91.24, and 93.22% of the initial capacitance after the 10,000 continuous GCD cycles, respectively. The corresponding Coulombic efficiency values are 98.66, 99.66, 98.80, 98.65, and 97.56% [23,30]. Based on the C_sp_ and R_ct_ values, the 5% Ni-doped SnO_2_ electrode studied in the RAE displayed an excellent electrochemical performance, suggesting that it is an appropriate electrode for energy storage applications.

## 4. Conclusions

Pristine and Ni-doped SnO_2_ nanoflakes were successfully synthesized by employing a simple template-free hydrothermal approach. The structural, morphological, compositional, and spectroscopic investigations confirmed the tetragonal structure of SnO_2_, and, additionally, the cubic structure of Ni as a Ni dopant introduced into the host SnO_2_ lattice. The effects of the Ni dopant in SnO_2_ have been revealed. A maximum C_sp_ of 841.85 F g^−1^ at 5 mV s^−1^ was measured for the 5% Ni/SnO_2_ in a KOH electrolyte. The faradaic pseudocapacitive profiles have been revealed from the electrochemical studies employing a KOH electrolyte and RAE. The impact of the RAE has been investigated. An ultra-high C_sp_ of 1335.77 F/g at 8 A/g was measured for the 5% Ni/SnO_2_ in the RAE, indicating an approximately 2.5-fold higher capacitance than in the KOH electrolyte. It is believed that this study will open new avenues for high-performance supercapacitors.

## Figures and Tables

**Table 1:** C_sp_ values from CV profile.

S. No	Scanning Rate (mV s^−1^)	C_sp (CV)_ (F g^−1^)				
		KOH Electrolyte				Additive Electrolyte
		Pristine SnO_2_	1% Ni/SnO_2_	3% Ni/SnO_2_	5% Ni/SnO_2_	5% Ni/SnO_2_
1	5	620.31	721.78	833.53	841.85	2130.33
2	10	570.75	653.96	791.42	799.84	1769.20
3	25	478.45	523.83	698.29	719.03	1415.07
4	50	407.52	400.90	605.71	648.73	1044.24
5	75	377.59	329.17	542.05	599.30	791.73
6	100	347.97	259.67	489.43	556.43	560.01

**Table 2:** C_sp_ value from GCD profile in KOH electrolyte.

S. No	Current Density (A g^−1^)	C_sp(GCD)_ (F g^−1^)			
		Pristine SnO_2_	1% Ni/SnO_2_	3% Ni/SnO_2_	5% Ni/SnO_2_
1	2	209.70	431.40	439.32	476.22
2	4	169.29	366.81	380.25	377.46
3	6	140.13	325.82	351.57	331.08
4	8	102.59	294.35	320.59	304.73
5	10	74.44	271.51	297.56	270.68

## Data Availability

Data and materials supporting the research are found within the manuscript. Raw data files will be provided by the corresponding author upon request.
